# Setting the stage for next-generation risk assessment with non-animal approaches: the EU-ToxRisk project experience

**DOI:** 10.1007/s00204-020-02866-4

**Published:** 2020-09-04

**Authors:** M. J. Moné, G. Pallocca, S. E. Escher, T. Exner, M. Herzler, S. Hougaard Bennekou, H. Kamp, E. D. Kroese, Marcel Leist, T. Steger-Hartmann, B. van de Water

**Affiliations:** 1 Leiden Academic Centre for Drug Research, Leiden University, Leiden, The Netherlands; 2 CAAT-Europe at the University of Konstanz, Constance, Germany; 3 Fraunhofer Institute for Toxicology and Experimental Medicine (ITEM), Hannover, Germany; 4 Edelweiss Connect GmbH, Basel, Switzerland; 5 German Federal Institute for Risk Assessment (BfR), Berlin, Germany; 6 Technical University of Denmark, Kongens Lyngby, Denmark; 7 BASF SE, Ludwigshafen, Germany; 8 TNO Innovation for Life, Utrecht, The Netherlands; 9 In Vitro Toxicology and Biomedicine, Department Inaugurated By the Doerenkamp-Zbinden Foundation at the University of Konstanz, University of Konstanz, 78457 Constance, Germany; 10 Investigational Toxicology, Bayer AG, Pharmaceuticals, Berlin, Germany

## Abstract

In 2016, the European Commission launched the EU-ToxRisk research project to develop and promote animal-free approaches in toxicology. The 36 partners of this consortium used in vitro and in silico methods in the context of case studies (CSs). These CSs included both compounds with a highly defined target (e.g. mitochondrial respiratory chain inhibitors) as well as compounds with poorly defined molecular initiation events (e.g. short-chain branched carboxylic acids). The initial project focus was on developing a science-based strategy for read-across (RAx) as an animal-free approach in chemical risk assessment. Moreover, seamless incorporation of new approach method (NAM) data into this process (= NAM-enhanced RAx) was explored. Here, the EU-ToxRisk consortium has collated its scientific and regulatory learnings from this particular project objective. For all CSs, a mechanistic hypothesis (in the form of an adverse outcome pathway) guided the safety evaluation. ADME data were generated from NAMs and used for comprehensive physiological-based kinetic modelling. Quality assurance and data management were optimized in parallel. Scientific and Regulatory Advisory Boards played a vital role in assessing the practical applicability of the new approaches. In a next step, external stakeholders evaluated the usefulness of NAMs in the context of RAx CSs for regulatory acceptance. For instance, the CSs were included in the OECD CS portfolio for the Integrated Approach to Testing and Assessment project. Feedback from regulators and other stakeholders was collected at several stages. Future chemical safety science projects can draw from this experience to implement systems toxicology-guided, animal-free next-generation risk assessment.

**Electronic supplementary material:**

The online version of this article (10.1007/s00204-020-02866-4) contains supplementary material, which is available to authorized users.

## Introduction

A particular focus of EU-ToxRisk is on endpoints for repeated-dose (RDT) and developmental and reproductive toxicity (DART), and on providing guidance for practical implementation (Daneshian et al. 2016). This entails the implementation of NAM-based safety science into regulatory toxicology (Krebs et al. 2020b). The project initially focused on read-across (RAx) (Escher et al. 2019; Rovida et al. 2020a) and here we outline key outcomes and general learnings on the use of NAMs for RAx. This collection of outputs will facilitate new efforts in next-generation risk assessment (NGRA) (Dent et al. 2018; Dearfield et al. 2017; Cote et al. 2016; Krewski et al. 2014) and thus advance the field of safety assessment by more mechanistically driven and animal-free approaches (Suppl. Box 1). This report was assembled once an important project phase came to its conclusion. We will accentuate especially learnings on the regulatory implementation of new approaches. Moreover, we highlight some gaps and unresolved issues that will need future attention.

## Incorporation of NAMs in read-across approaches

The main activity of the project addressed the application of NAMs to support the most widely applied method of data-gap filling: RAx. RAx may support safety assessment for the complex toxicological endpoints RDT and DART, yet it often fails to be accepted by regulatory agencies. Reasons for rejection often include the notion that associated uncertainties are perceived as being too large. Classical RAx is based on structural similarity as the major argument, and there are many examples that chemical formula alone does not sufficiently predict toxicity. This uncertainty can be substantially reduced when NAMs demonstrate that source and target chemicals share similar biological and toxicokinetic profiles, providing justification for reading across source chemicals’ in vivo data.

**Fig. 1:**
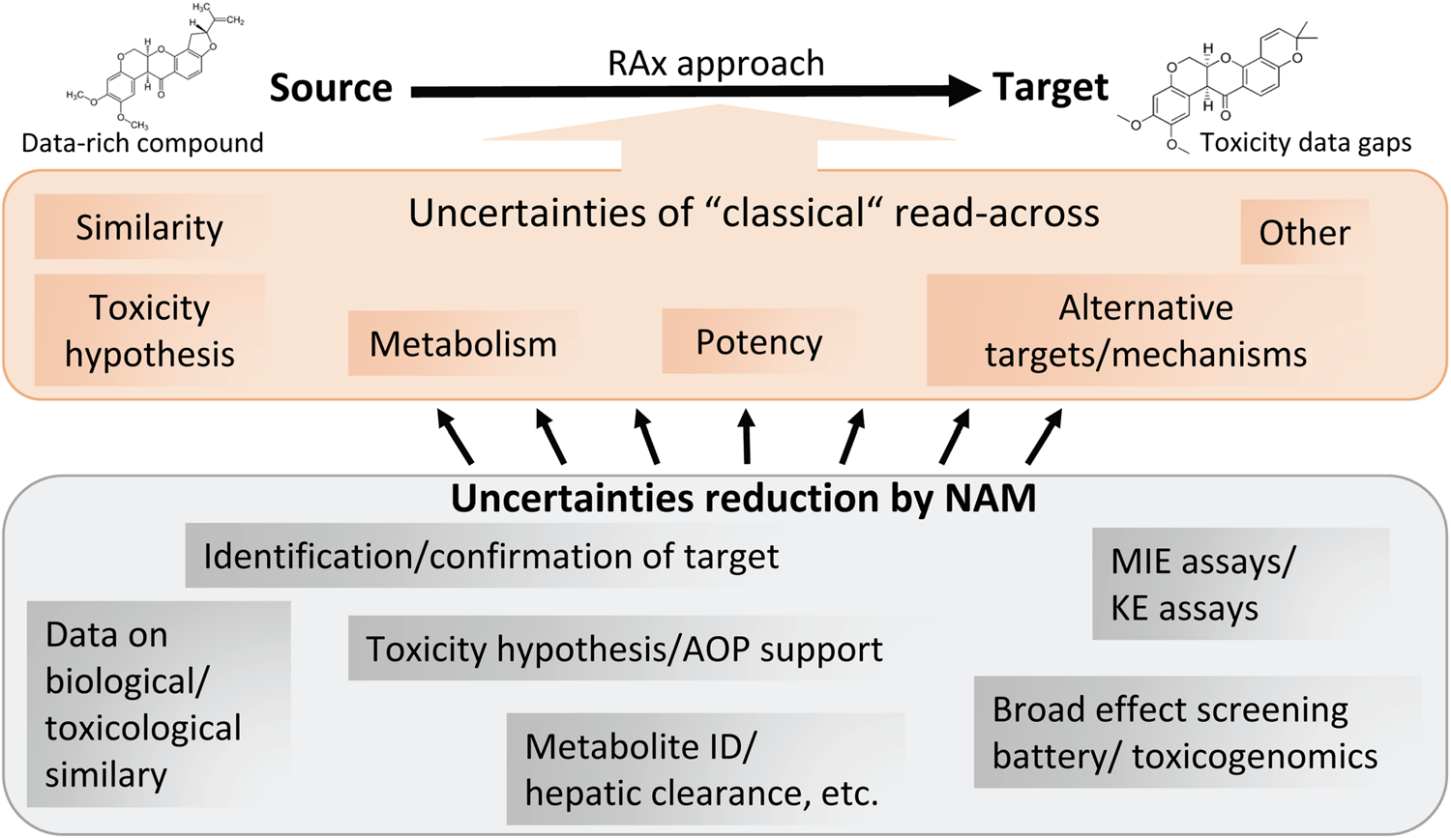
Reduction of RAx uncertainties by NAMs. The top box lists several uncertainties that may weaken a RAx approach (e.g. uncertainty on the similarity of source and target, uncertainty on metabolism, or uncertainty concerning the potency ratio of source and target). The bottom box indicates information that can be provided by NAMs to reduce uncertainty (e.g. data on potency ratios in key event (KE) or molecular initiation event (MIE) assays, or the identification (ID) of relevant metabolites formed)

The project hypothesis was that NAMs can reduce uncertainty in traditional RAx (Fig. 1). For instance, they help characterize the biological properties of source and target compounds. NAMs can point out a specific molecular initiation event [e.g. receptor (ant)agonism], provide data on test compound hazard (i.e. expected types of adverse outcomes), elucidate a mode of action (i.e. pathways and targets affected), or assess relative potencies of the observed effects. Also, the absence of a certain mechanism or effect can be demonstrated, as well as the relatively low potency for a certain testing endpoint. The EU-ToxRisk RAx testing framework demonstrated how NAMs can be integrated into practical RAx procedures (Escher et al. 2019).

If the AOP for a set of chemicals is known, NAM testing can be designed along this mechanistic framework to explore molecular initiating events (MIEs) or key event (KE) responses. Targeted testing aims to generate mechanism-related data for all grouped compounds, to either confirm (dis)similarity or observe a consistent trend. If no AOP is known, the battery of NAMs can be chosen in a way to capture as many as possible potential underlying mechanisms. NAMs can alternatively be used for broad untargeted testing to either (1) generate a RAx hypothesis based on shared in vitro effects, or (2) to prove the absence of effects (up to concentrations corresponding or exceeding those obtained in humans under realistic exposure situations).

Besides resolving many conceptual uncertainties, NAM data, like any other type of experimental result, may introduce their own uncertainty (assay variations, random noise, and congruence of different tests). This issue can be addressed by applying a structured and weighted description of uncertainties. This may use generalized Bayesian approaches or Dempster–Shafer decision theory (Dempster 1967; Rathman et al. 2018; Shafer 1976), or it may use semi-quantitative approaches based on expert judgment (Escher et al. 2019; Schultz et al. 2018). For instance, the Dempster–Shafer approach allows for a fully quantitative combination of various types of test data, taking into account the individual test performances/uncertainties, and to derive likelihoods of test data being correct. EU-ToxRisk puts significant emphasis on diligent uncertainty descriptions and in taking initial steps towards uncertainty quantification. More resources will need to be put in place to apply these concepts in a more routine manner, and the science of uncertainty assessment is likely to become an important support discipline of risk assessment.

## Brief overview of case studies

Some of the project CSs were selected to test regulatory applicability of NAM-supported RAx. The design of a case study implied (1) the definition of a target chemical and a regulatory question, and (2) the delineation of data gaps and the approaches to fill them. A detailed overview and flowchart have been presented elsewhere (Graepel et al. 2019; Escher et al. 2019).

CSs were based on assemblies of structurally similar compounds to demonstrate how NAMs can be used to substantiate a RAx hypothesis. CSs always contained some chemical analogues with in vivo endpoint data, so that predictivity and accuracy of the NAM data could be verified. For the same reason, these CSs also included structurally similar compounds that did not exhibit a shared toxicological effect patterns/AOPs in the in vivo data determining the RAx hypothesis. NAM data were subsequently used to better define the boundaries of the categories and to reduce uncertainties. The description of the individual CSs is beyond the scope of this editorial and will be highlighted in individual publications. Here we specifically address the overall learnings and impact of the CSs for RAx and their relevance for future next-generation risk assessment.

To illustrate the CS concept, we will describe a typical example: a CS focusing on the substance deguelin as a potential inducer of parkinsonian motor deficits. An AOP-based testing strategy was applied to support the RAx hypothesis. The AOP for inhibition of mitochondrial complex I of nigrostriatal neurons leading to parkinsonian motor deficits (Terron et al. 2018) was used to assess the biological similarity of two different rotenoids: rotenone (source compound, data-rich) and deguelin (target compound, data-poor). For the source compound, there was compelling evidence for the induction of Parkinson’s-like disorders in experimental models, and also epidemiological evidence for similar effects in human (Ntzani et al. 2013). Various NAMs were selected to represent the MIE and KEs of the AOP. Both, in silico and in vitro test methods were applied. For instance, in silico structural modelling was used to identify the common pharmacophore that determines the binding to complex I, being the MIE of the AOP. To determine effects on the KEs, several in vitro methods were used to detect effects on mitochondrial respiratory activity (oxygen consumption) and on proteostasis. High-content imaging methods were applied for measuring damage to human dopaminergic neurons, and to compare the effects of the two compounds. Finally, PBK modelling was performed to compare biokinetics and distribution of both compounds to the brain. The approach chosen in this case study demonstrated that an AOP-based testing strategy combining different test methods that cover the various KEs can be applied in supporting chemical safety assessment within a NAM-based RAx framework.

## Feedback by the Regulatory Advisory Board

Given the overall EU-ToxRisk objectives to provide solutions for implementing NAMs in risk assessment, the NAM-enhanced RAx case studies were presented and discussed on several occasions for feedback from the regulatory community. For this purpose, the project’s Scientific Advisory Board initiated close collaborations with regulators from national, European and international regulatory authorities, including BfR and RIVM, ECHA, EFSA and EMA, and NIEHS, respectively. This cooperation resulted in an improved mutual understanding of the requirements and pitfalls of RAx approaches supported by NAMs, from a scientific, academic, and regulatory perspective. The project’s Regulatory Advisory Board (representing the main agencies/authorities) was set up in the course of the study as one of the first reactions to stakeholder feedback. It has since been key for the establishment of a reporting template for RAx cases. The extensive documentation and pre-validation of the EU-ToxRisk NAM toolbox is a step forward to increase the acceptability of regulatory dossiers based on NAM data. Overall regulatory learnings are summarized in Box 1.

Box 1: Regulatory learnings based on the NAM-based RAx framework assessment I. Involve regulators from the start in the design of CSs so that they revolve around precisely phrased regulatory questions.

II. Meticulously define both regulatory context and hypothesis.

III. Provide a rationale for each step in the risk assessment argument that regulators can follow and thus evaluate.

IV. Provide a clear learning scenario. Sharply define the situation before the RAx and before the use of NAMs. Evaluate the situation afterwards and provide transparent conclusions of what each method contributed.

V. Compare established risk assessment approaches with NAM-based RAx approaches and identify advantages and shortcomings together with needs for future implementation training.

VI. Close interaction with the wider community of regulatory stakeholders is crucial to the establishment of the procedures. “Getting the science right” is very important, as is “getting the framing and reporting right” to match expectations and needs of the regulatory target audience.

## Collected feedback from stakeholders and key opinion leaders on the EU-ToxRisk NAM-supported RAx framework

Following internal regulatory scrutiny, the project CSs and approaches were discussed in external forums. Learnings, achievements, and pitfalls of the approach were also analysed in detail in a dedicated workshop organized by EU-ToxRisk in May 2019 in Espoo, Finland. Over 60 international experts from industry, academia, and regulatory authorities explored five scientifically advanced project case studies developed within EU-ToxRisk, the OECD/Integrated Approach to Testing and Assessment (IATA) program, and NIHS Japan. The wide range of expertise and perspectives of the participants yielded valuable insights and answers to where and how NAMs can effectively support a RAx problem formulation, identify areas that contain data gaps, suggest how these gaps can be filled, and finally provide technical guidance on how those steps should be presented in a RAx regulatory dossier. Reports of three of the most mature EU-ToxRisk case studies, describing the RAx question, the approach taken (i.e. what NAM to select), the results obtained, and the conclusions derived, were further shared with the regulatory community for their review and feedback (Box 2).

Box 2: Learnings from stakeholder feedback *Scientific*

I. Relevant aspects, such as xenobiotic metabolism and chemical or biological similarity considerations, should be addressed all at the same time to ensure a holistic coverage of potential contexts. Too much focus on one particular similarity context bears the danger of missing/neglecting important effects and relevant context.

II. The regulatory question is important for determining the scope of the scientific approach. In some cases, all potential toxicities (or metabolites, or aspects of the chemical structure) may need to be considered. In other cases, it may be well acceptable to focus only on one relevant toxicity (e.g. neurotoxicity) or on one specific toxicophore.

III. Anchoring a toxicity hypothesis to an AOP is desirable. If not available, one may still perform a weight-of-evidence approach using, for instance, a battery of NAM tests.

*Regulatory*

I. Qualitative expression of uncertainty is to be done in explicit categories (low/medium/high). Non-quantifiable uncertainties have to be made transparent. All underlying uncertainties of the assessment need to be covered, including category formation, metabolism predictions, choice of NAMs, etc.

II. Focus and limit testing. The goal should be to do as much testing as needed to achieve sufficient confidence in a RAx justification, but not more than that.

## Case study reviews by the OECD IATA Case Studies Project group

In parallel, these same three project case studies plus an additional one were also submitted to the OECD for review in its 5th Meeting of the IATA Case Studies Project under the remit of the Working Party on Hazard Assessment. The IATA Case Studies Project aims to assess the practical applicability of NAMs, as a part of the IATA framework, for different aspects of regulatory decision-making to build assessment experience (OECD 2015). The submitted EU-ToxRisk project CSs underwent rigorous review by risk assessors from several countries, the OECD, and the International Council on Animal Protection in OECD (ICAPO). The reviewing had a broad scope, assessing not only the data but also identifying where further guidance would be helpful, e.g. the regulatory relevance and the reporting. The OECD consideration document is set to be published adjacent to the CSs after their finalization based on comments received. Final endorsement by the Working Party on Hazard Assessment and the Joint Meeting members allows for their publication in September 2020. This represents a major milestone toward regulatory recognition of NAM-based RAx (Box 3).

Box 3: Learnings from OECD feedback *Scientific*

I. Provide argumentation for the selection of all compounds and the rationale for their selection (or exclusion).

II. It is advantageous to quantitatively extrapolate NAM-based data to the (human) in vivo situation.

III. A good toxicity hypothesis (e.g. AOP-based) supported by NAM data can serve as justification for RAx.

IV. Inclusion of high-content data (e.g. toxicogenomics) can increase overall confidence (e.g. of not missing important adverse effects).

*Regulatory*

I. Due to the lower level of validation of and experience with many NAMs in a regulatory context, it has to be ensured that recipients of RAx reports can understand all data provided, especially when methods are more complex and less well described in the literature.

II. Strengths and weaknesses, as well as rigorous uncertainty assessment, should be clearly addressed in the reporting.

## EU-ToxRisk advisory document on the proposed read-across framework

From the three different case study reviewing phases, it became evident that RAx justifications should be presented as logically structured, coherent “stories”, along the “as comprehensive as needed, as concise as possible” principle.

Here the need for an advisory document that would be complementary to RAAF (ECHA 2017) and the OECD guidance document for in vitro test method descriptions (OECD 2014), addressing regulatory acceptance from the point of view of registrants of NAM-supported RAx dossiers. The Read-Across Advisory Report was structured back-to-back with the EU-ToxRisk overall RAx framework (Escher et al. 2019) and includes common learnings from the above discussion.

The advisory document targets the broader toxicology community with practical instructions on the applications of NAM-based RAx in different regulatory contexts, including all relevant collected feedback and endorsements on taken approaches. Its application will improve the submission quality of RAx cases by registrants and thereby increase successful acceptance rates of non-animal approaches. For this purpose, an important component of the template already now is guidance on how to fill out the different sections of the risk assessment reporting submission document—especially with NAM data. A web-based graphical user interface (GUI) is being produced to facilitate its use.

## The repository of NAMs available for NAM-enhanced RAx

**Fig. 2:**
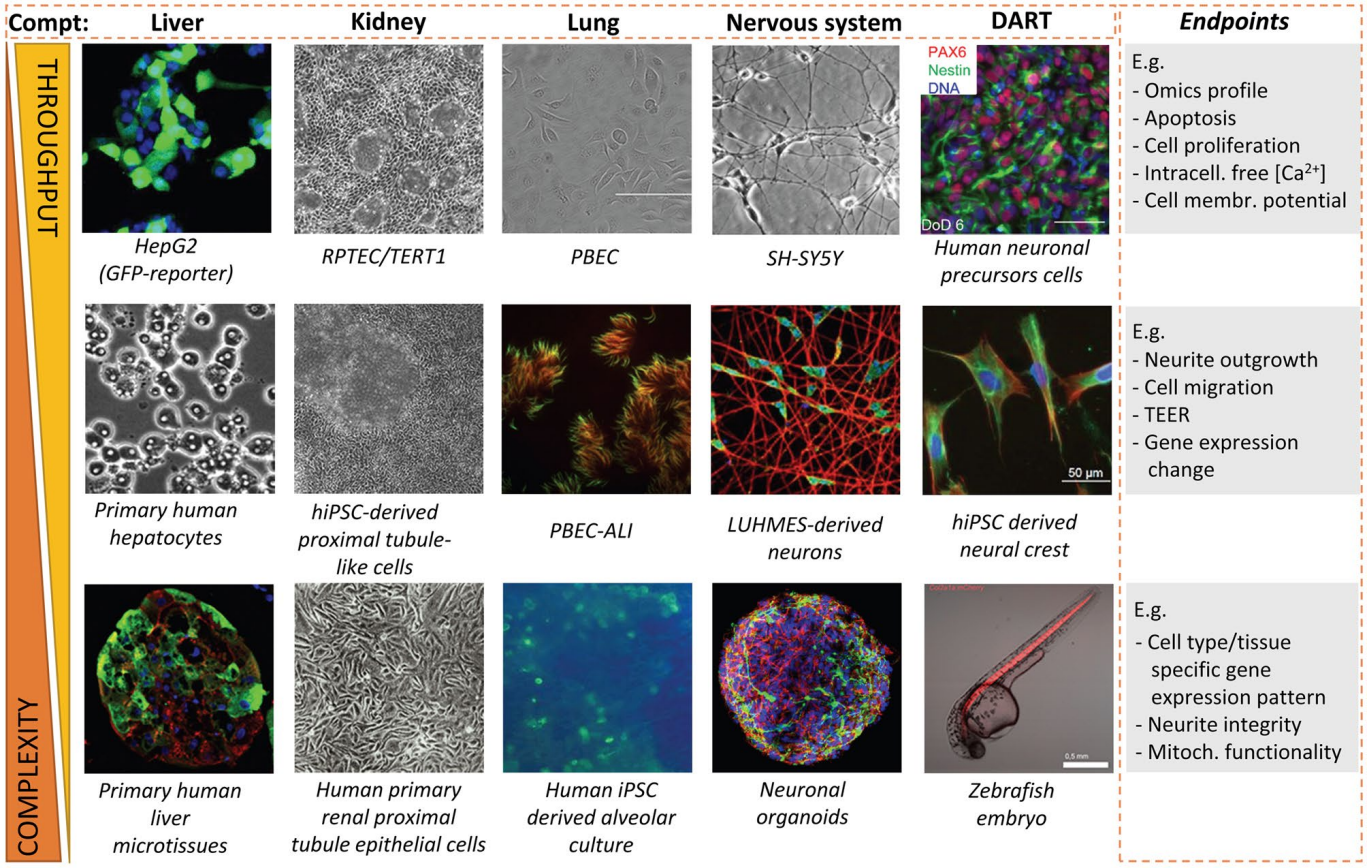
The EU-ToxRisk method toolbox. The EU-ToxRisk method toolbox includes test systems using cells from four major organ systems. In addition, models allowing readouts on DART have been included. The toolbox comprises both simpler cell models (2D, monoculture, etc.) and complex systems (3D, co-cultures, zebrafish embryos). The characteristics of each model determine their throughput and their use at different stages of case studies. For each test system, various endpoints have been established, so that assays can be run to assess effects on cell viability in parallel with functional, biochemical, and toxicogenetics endpoints. Figure adapted from the EU-ToxRisk project’s website (https://www.eu-toxrisk.eu/page/media_items/test-methods8.php)

EU-ToxRisk started off with many NAMs available at partner laboratories. They were selected and refined with the purpose of ensuring applicability in different regulatory contexts. Based on the initial toolbox, existing test methods were continuously refined to enhance their applicability based on two parameters: test system complexity and test method throughput (Fig. 2). Simpler test systems were combined to obtain advanced complex test systems to better resemble the in vivo situation. These approaches include, for instance, the use of mixed cell cultures (Gutbier et al. 2018), 3D organoids and neurospheres (Brull et al. 2020; Hiemstra et al. 2019; Kobolak et al. 2020) and microphysiological systems (MPS), e.g. four-organ-chips developed to interconnect miniaturized human intestine, liver, brain and kidney equivalents (Ramme et al. 2019) and improved iPSC cell differentiation to hepatocytes (Boon et al. 2020). These advancements were fuelled by the establishment of novel differentiation protocols that produce high-quality cellular test systems (Ballester et al. 2019; Coll et al. 2018; Dreser et al. 2020; Gu et al. 2018; Rauch et al. 2018).

In parallel, the availability of advanced high-throughput approaches was exploited to increase the applicability domains and throughput levels of the test methods. Screening transcriptomics data were provided from hepatic (Albrecht et al. 2019; Campos et al. 2020; Copple et al. 2019; Ramirez et al. 2018), renal (Limonciel et al. 2018a, b) and neuronal (Delp et al. 2019) cell systems. High-content imaging was expanded to complex toxicological endpoints, such as DART (Dreser et al. 2020; Nyffeler et al. 2018) and systemic RDT with the establishment of novel fluorescent protein reporter cell lines (Bischoff et al. 2019; Schimming et al. 2019; Wink et al. 2018; Yang et al. 2020).

The toolbox was further enriched by novel in silico approaches, supporting and complementing in vitro cell-based NAMs. An important area here was the extrapolation of NAM concentrations to in vivo doses (IVIVE, PBPK) (Fisher et al. 2019; Simeon et al. 2020; Toma et al. 2018). Additionally, novel QSAR tools and machine learning approaches were put in place to assess chemical similarity among test compounds (Gadaleta et al. 2018a, b; Hemmerich et al. 2020; Luechtefeld et al. 2018; Toropova et al. 2018; Toropov and Toropova 2017; Troger et al. 2020) and to predict toxicological properties from their structure.

Finally, the project defined new AOPs to seamlessly integrate data generated by alternative methods or in vivo testing in a mechanistic and quantitative manner (Maertens et al. 2018; Terron et al. 2018; Zgheib et al. 2019).

All this work contributed not only to the regulatory implementation of NAM-enhanced RAx, but also presented a scientific advance recognized by the academic community. This output is exemplified here by a selection of publications according to toxicological topics (Supp. Table 1), and high-ranking lists of papers in terms of citation and journal impact factor (Supp. Table 2; Supp. Table 3).

## Knowledge infrastructure: sharing of data, test methods, results and knowledge

The scientific progress described above opens up new possibilities for reducing uncertainties associated with RAx and other risk assessment approaches. Irrespective, there was valuable feedback from regulatory specialists on the relative importance of high-end science vs the more basic quality control and documentation aspects. An important notion was that it is critical for regulators to quickly understand a new method; its information value, its purpose and its quality status. Similar considerations applied to data generated via NAMs, and it was enlightening to many contributing partners that the level of information sufficing for publication did not scale with regulatory requirements. The project team responded to this essential notion from the Regulatory Advisory Board by ensuring that all NAMs are described in a maximally transparent way to enable regulatory toxicologists to assess quality, reliability, applicability, and relevance of method outputs (Pamies et al. 2020; Bal-Price et al. 2018; Krebs et al. 2019). This approach was addressed by the use of comprehensive test method descriptions that also allow for the assessment of readiness levels of individual tools, combined with an efficient procedure for data upload (Krebs et al. 2020b). An efficient data management system was developed as a hub to transparently enable connecting methods to data, integrating data derived from different methods, and linking the integrated data to confirm or reject an overall testing hypothesis (like RAx).

In the context of a cross-system testing case study, detailed protocols for FAIR data handling were established, including the provision of sufficiently rich and transparent metadata explaining how data was produced. This includes the experimental setup and the various processing steps from raw to summary data such as benchmark concentration (Krebs et al. 2020a) or no-observed-adverse-effect-levels (NOAELs) used in risk assessment. EU-ToxRisk, together with its stakeholders, created test method descriptions and provided guidance on data processing (Krebs et al. 2018). A knowledge infrastructure was programmed that allows for efficient management of this information, using structured but still flexible input. The starting point is a detailed test method description including a standard operating procedure (SOP), and also more specific details on the validation status and relationship of the assay to AOPs.

The developed test method documentation (ToxTemp) covers mostly transparency and validation aspects. It was designed in alignment with the OECD Guidance Document 211, which provides a template for “descriptions of non-guideline in vitro methods”. The many types of essential information can be grouped into four sections: (1) the overall test method description, (2) the technical test procedure (as outlined in an SOP, e.g. defined labware, consumables and pipetting steps), (3) the characterization of test and reference materials/chemicals, and (4) all issues relating to data processing and archiving. Finally, additional paragraphs address the test purpose, the test limitations (like information on its applicability), and the criteria to be used for interpreting test results. ToxTemp was endorsed by the project’s external advisory boards and over 30 experts from industry, regulatory bodies, and academia (Krebs et al. 2019). The public version of the ToxTemp method repository is available at https://eutoxrisk.edelweissconnect.com/public/). It is additionally used to provide metadata for the associated datasets as computer-readable annotations.

**Fig. 3:**
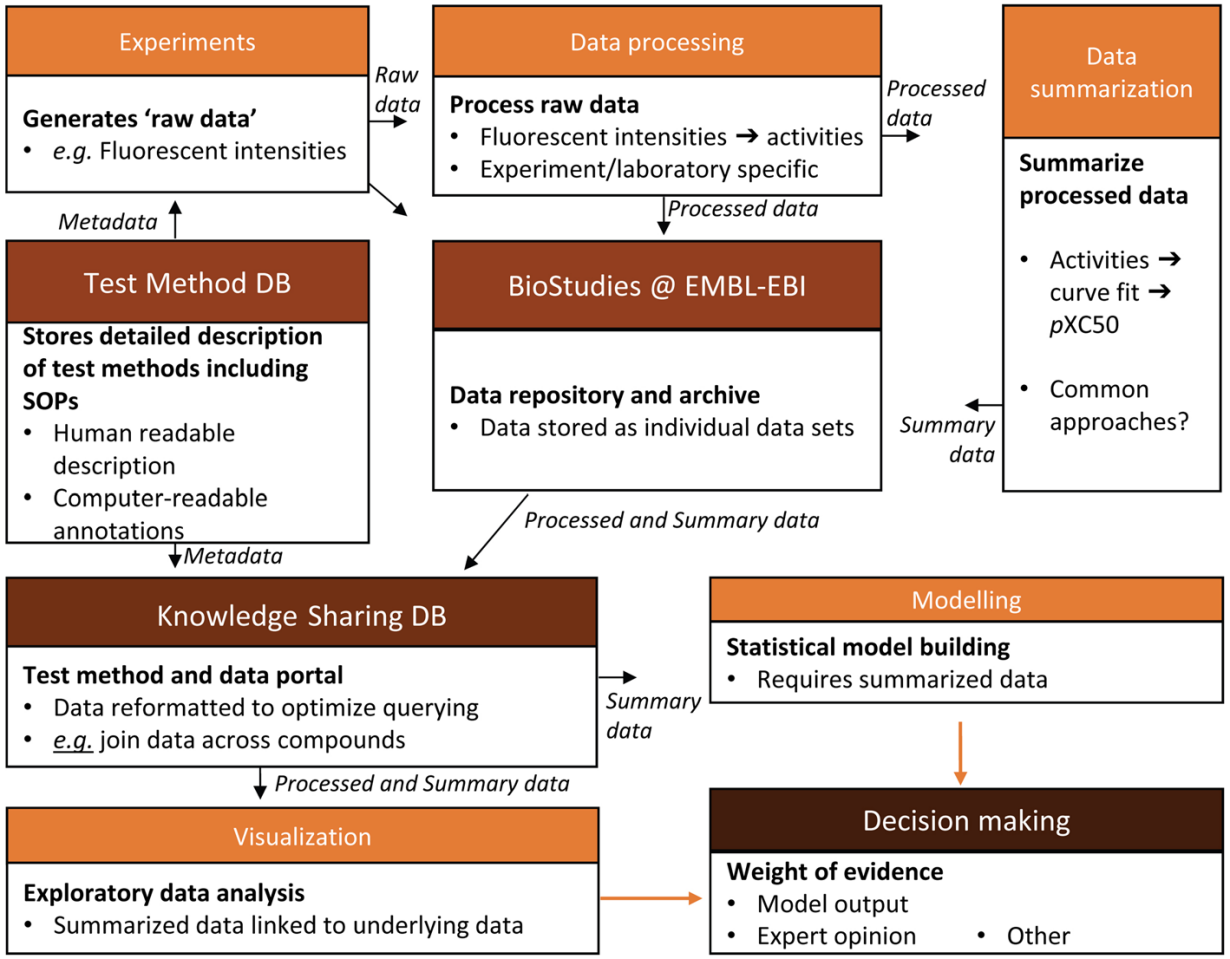
The EU-ToxRisk knowledge-sharing infrastructure. Data and metadata follow different flows to be processed and deposited into the respective data infrastructure. Raw data are processed and summarized before being deposited into the Biostudies database at EMBL-EBI. Metadata are generated for each data set and deposited together into the knowledge-sharing database run by Edelweiss Connect. The coupled information is used for statistical model building and can be explored via visualization tools. The collection of information allows for weight-of-evidence-based decision-making processes

All EU-ToxRisk metadata linked to all project data output will become available from the BioStudies database at the European Bioinformatics Institute (EBI) once the data have been publicly released (Sarkans et al. 2018). The EU-ToxRisk knowledge-sharing platform accesses data from there and provides it to the consortium in a structured way for searching, browsing, visualization, and modelling purposes and, ultimately, for decision-making in risk assessment as exemplified in project case studies (Fig. 3). Collecting test method information and metadata can be a time-consuming and error-prone task if done manually. Therefore, automated procedures were designed to collect data and metadata from experimental equipment and processing software and provide automated file validation tools to check data consistency, complemented by human curation by multiple individuals as a final quality check (Box 4).

Box 4: Learnings on data management I. Use harmonized templates for data and methods right from the project onset.

II. Invest time and resources into early training of researchers on data handling and management.

III. Provide and flexibly adapt data conversion and input tools.

## Gaps and future requirements

Despite significant progress, there are still gaps that need to be addressed before standard risk assessment can be performed entirely with animal-free methods. A comprehensive gap analysis would require an extensive vision document on the future of toxicology. This is beyond the scope of this short overview, but for an overview of past and current ideas, we refer to various visionary reports and documents (Cote et al. 2016; Hartung and Leist 2008; Khadka et al. 2020; Krewski et al. 2020; Leist et al. 2014, 2008; Lupu et al. 2020; Sauer et al. 2015; Thomas et al. 2019, 2018).

**Fig. 4:**
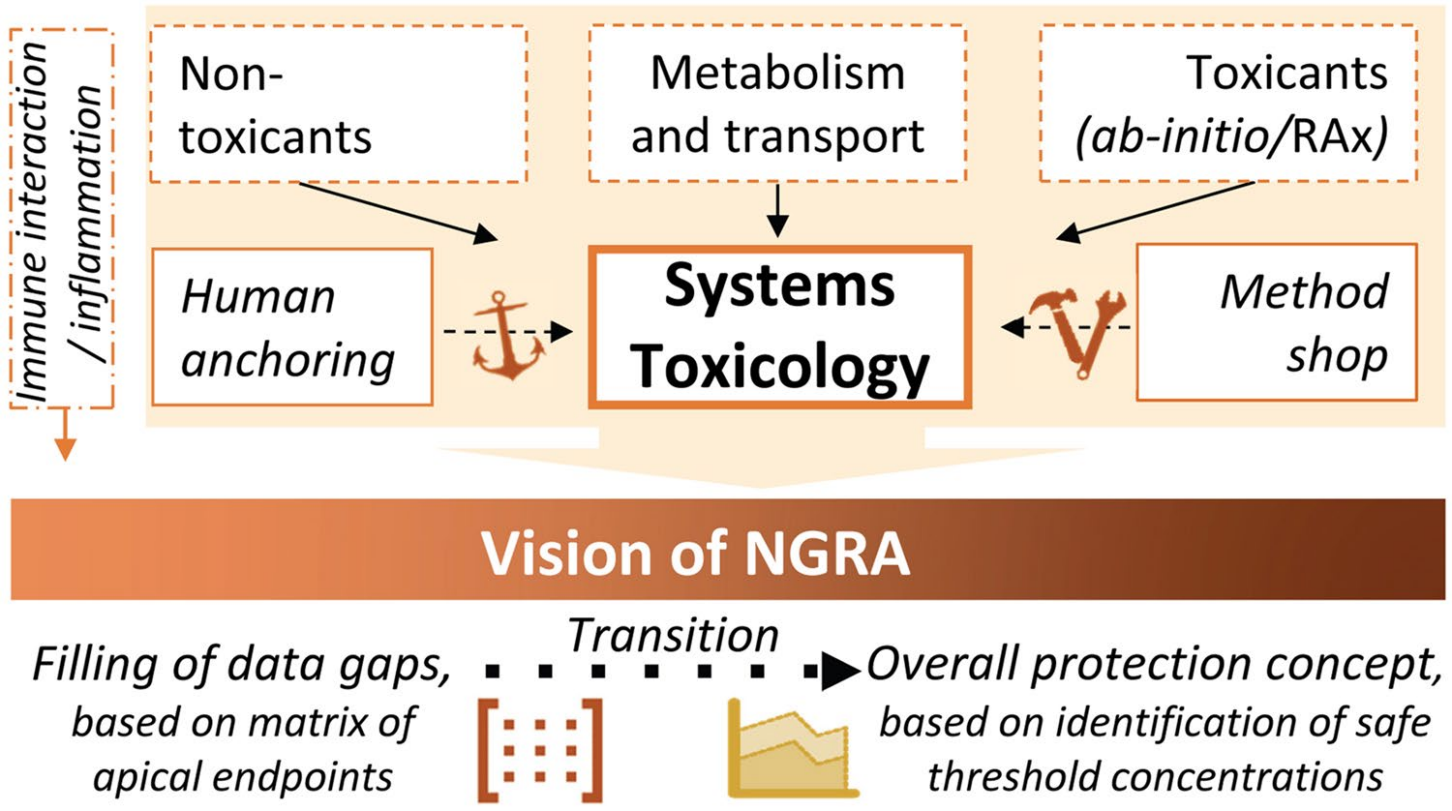
Elements of next-generation risk assessment (NGRA) framework. NGRA should see the shift from mainly using NAMs for filling demarcated data gaps to a human-centric overall protection concept underpinned by NAM-based hazard quantification. Immune responses need to be taken into account to predict idiosyncratic reactions and chronic health consequences. To quantify risk and while accounting for associated uncertainties, NGRA will rely on integrative systems toxicology-based modelling approaches, anchoring safety testing to in vivo human biology. Considering metabolism and transport early on will be key to arrive at hazard scenarios for new chemical compounds. To cater for the wider field of applied toxicology, NGRA should be developed with both toxic and non-toxic, and both data-rich and data-poor compounds in mind

Here, we outline nine key aspects that need further attention. This itemized summary may help in structuring work packages and defining strategies of future large risk assessment projects (Fig. 4):

1. *Method shop* The width of in vitro and in silico NAMs—and of strategies using them—is very large compared to traditional animal models. New concepts and business models are required to make broad panels of new models available to all stakeholders.
2. *Human anchoring* Method validation has traditionally relied on the correlation of NAM data with animal study data. Even though animal data was useful for the development of NAMs, they also have shortcomings towards chronic/long-term human health consequences. Therefore, the development and calibration of NAMs should also be anchored to human (patho)biology and not be calibrated exclusively by animal data.
3. *Metabolism and transport* More quantitative methods are required to predict the influence of metabolism and transport on the hazard of novel compounds.
4. *Ab initio assessment* Strategies need to be worked out to quickly arrive at a mechanistic hypothesis for the potential hazard of compounds with little or no prior safety information.
5. *Non-toxicants* A comprehensive strategy needs to be established on how to define compounds of low or no toxicity. This also requires measures for uncertainty. In particular, the risk of false-negative assessment needs to be evaluated.
6. *Immune system* Both native and adaptive immune responses can be involved in toxicity (Benedetti et al. 2013; Fasbender et al. 2020; Fredriksson et al. 2011, 2014; Leist et al. 1997; Monshi et al. 2013). This notion needs further exploration to predict idiosyncratic reactions and chronic health consequences for more susceptible individuals.
7. *Protection perspective* Various past efforts attempted to substitute traditional hazard assessment with modern NAMs, following the concept that defined adverse events need to be identified and the subsequent animal-based method to be substituted one-on-one by NAM(s). This strategy will fail for complex endpoints. It is even doubtful whether such an approach can cover all potential target organs and tissues in the human body, be it practically or at an acceptable cost. A future safety science concept may require a more revolutionary approach that is not anchored to traditional adverse outcomes, but rather to the identification of safe concentrations. Staying below such concentrations would protect the population from toxic effects by ensuring that no biological events linked to toxicity are activated. While some CSs of EU-ToxRisk started exploring this concept, future projects need to address this systematically.
8. *Systems toxicology* Points 3–6 need to be successfully incorporated into a comprehensive systems toxicology model, which will then eventually form the basis for point 9. This will involve outputs not only on concentration thresholds associated with hazard (and extrapolations to respective doses/exposures), but also quantify the associated uncertainties.
9. *Next-generation risk assessment* Similar to the work of EU-ToxRisk on hazard assessment, the next step is to progress towards NGRA, using NAM-based hazard quantification in combination with defined exposure scenarios and exposure models.

An important future step to increase the impact of EU-ToxRisk approaches will be the uptake of its tools and strategies in upcoming initiatives (e.g. the Horizon Europe Partnership for the Assessment of Risk from Chemicals; PARC) and in collaboration with the private sector. Already some CSs in partnership with external industry stakeholders were initiated. Such joint CSs help to (1) inform strategic decisions, (2) prioritize chemicals within a group, and (3) support the problem-solving process in investigative toxicology (Beilmann et al. 2019). Furthermore, the sustainability of the approaches will be supported by a commercialization platform that consolidates NAMs and NAM data from different partner organizations, and use the integrated results in both safety assessment and investigative toxicology. In this context, activities aiming to increase the international acceptance of the NAMs are crucial. This requires continued interactions amongst all relevant stakeholders groups, including industry, regulatory agencies, academia, policy-makers, and NGOs (Busquet et al. 2020). For this reason, EU-ToxRisk provided many discussion platforms in the form of stakeholder meetings, conference sessions, and closed workshops with regulators and parallel projects. Recent examples of such activities are the workshops on microphysiological systems and their regulatory application (Marx et al. 2020) and on international acceptance of read-across approaches (Rovida et al. 2020a, b) (Box 5).

Box 5: General learnings for future risk assessment projects: I. Establish a Regulatory Advisory Board right from the onset (application phase).

II. Structure the project along CSs rather than work packages.

III. Anchor each CS to a strictly defined regulatory question.

IV. Use fit-for-purpose and well-structured risk assessment reporting templates (compatible with regulatory requirements).

V. Ensure a strong emphasis on data management and establish consensus method description templates and data upload templates and provide a project data integration platform.

VI. Build and maintain networks with other toxicology programs. Maintain good personal relationships. Joint events and activities are necessary to make exchanges work, so reserve budget and resources to this end.

VII. For projects with a long runtime (≥ 5 years), allow for some re-organization possibilities and the re-alignment of scientific goals as technologies progress and regulatory practice evolves.

VIII. Reserve budget for test method validation and sustainability measures.

IX. For all CSs, establish starting knowledge (pre-registration); then evaluate the gain of knowledge after CS finalization.

X. For larger projects, finance and recruit a full-time scientific manager (in addition to administrative management and overall project coordination).

## Electronic supplementary material

Below is the link to the electronic supplementary material.

Supplementary file1 (PDF 924 kb)
