# Supplementary material for: Setting the stage for next-generation risk assessment with non-animal approaches: the EU-ToxRisk project experience
Source: Arch Toxicol. 2020 Sep 4;94(10):3581–92. doi: 10.1007/s00204-020-02866-4 (PMC7502065; doi:10.1007/s00204-020-02866-4)
Supplement: Supplementary file 1 — Supplementary file1 (PDF 924 kb) [file 204_2020_2866_MOESM1_ESM.pdf]

Supplementary material

Setting the stage for next-generation risk assessment with non-animal approaches: the EU-ToxRisk project experience

Moné MJ[1], Pallocca G[2*], Escher SE[3], Exner T[4], Herzler M[5], Hougaard Bennekou S[6], Kamp H[7], Kroese ED[8], Leist M[2,9], Steger-Hartmann T[10], van de Water B[1]*

[1] Leiden Academic Centre for Drug Research, Leiden University, Leiden, The Netherlands
[2] CAAT-Europe at the University of Konstanz, Konstanz, Germany
[3] Fraunhofer Institute for Toxicology and Experimental Medicine (ITEM), Hannover, Germany
[4] Edelweiss Connect GmbH, Basel, Switzerland
[5] German Federal Institute for Risk Assessment (BfR), Berlin, Germany
[6] Technical University of Denmark, Kongens Lyngby, Denmark
[7] BASF SE, Ludwigshafen, Germany
[8] TNO Innovation for Life, Utrecht, The Netherlands
[9] In vitro Toxicology and Biomedicine dept, University of Konstanz, Konstanz, Germany
[10] Investigational Toxicology, Bayer AG, Pharmaceuticals, Berlin, Germany

Supplementary Box 1: Background information on Next Generation Risk Assessment (NGRA)……………..p. 2

Supplementary Table 1: Selected EU-ToxRisk publications…………………………………………………..…………….p. 3

Supplementary Table 2: Top-10 EU-ToxRisk publications ranked by citation rate……………………………..……p.11

Supplementary Table 3: Top-10 EU-ToxRisk publications ranked by journal impact factor………………….p.13

Supplementary references……..…….p.15

Supplementary Box 1: Background information on Next Generation Risk Assessment (NGRA)

Next-generation risk assessment (NGRA) is a loosely-defined term with several roots. A major driving force has been the report of the national academy of sciences of the USA of 2007, in which a roadmap for a toxicology of the 21st century was given (Leist et al. 2008; Leist et al. 2012). Various groups and consortia further promoted this idea (Collins et al. 2008; Tice et al., 2013; Leist et al. 2014; Pastoor et al., 2014; Embry et al., 2014; Gocht et al., 2015; Cote et al., 2016; Daneshian et al., 2016; Birnbaum et al., 2016; Juberg et al., 2017; Paini et al., 2019; Wolf et al., 2016). Major features are (i) the use of NAM to derive hazard and toxicokinetics data, (ii) the use of integrated approaches to testing and assessment (IATA), i.e. carefully considering all available information in addition to the exposure situation/problem formulation, (iii) the use of modelling approaches on the level of the individual exposed, but also on the level of the population at risk.

Supplementary Table 1: Selected EU-ToxRisk publications

Category	Author and Year	Title	Description
Advanced DART models	Dreser et al., 2020	Development of a neural rosette formation assay (ROFA) to identify neurodevelopmental toxicants and to characterize their transcriptome disturbances	In the area of DART, the publication describes the establishment of a human stem cell-based test to detect development neurotoxicity. The original test method was based on the detection of transcriptome changes. Now, this endpoint has been anchored to a functional read-out, the ability of toxicants to interfere with the normal capacity of neural precursors cells to self-organize to neural rosettes. Several established toxicants (like valproic acid) led to distinctly different tissue organization and differentiation stages that can be quantitatively measured.
Advanced RDT models	Gutbier et al., 2018	Prevention of neuronal apoptosis by astrocytes through thiol-mediated stress response modulation and accelerated recovery from proteotoxic stress	An advanced organ-specific assay was established and described. A well-established model of postmitotic human dopaminergic neurons (LUHMES cells) was used in the absence or in co-culture with astrocytes to investigate the mechanisms involved in the endogenous neuro-defensive activity of astrocytes. The presence of astrocytes attenuated the neuronal stress response increasing neuronal resilience to various proteotoxic stressors. Such knowledge not only applies to toxicological predictions but may also allow boosting of the brain's own defence mechanisms and could be used to increase its resilience towards toxicants or the progression of neurodegenerative disease.
	Hiemstra et al., 2019	High-throughput confocal imaging of differentiated 3D liver-like spheroid cellular stress response reporters for identification of drug-induced liver injury liability	The publication describes a novel adaptation to the already established HepG2-based fluorescent protein reporter platform. Such a platform was established to monitor adaptive stress response activation following DILI drug treatment. The test system was now improved to increase its metabolizing capacity by the use of 3D liver-like spheroid cultures. This new approach was challenged with several chemicals. The results indicate that the test system is a promising tool for mechanism-based identification of compounds with liability for DILI.
	Ramme et al., 2019	Autologous induced pluripotent stem cell-derived four-organ-chip	The authors presented a four-organ-chip developed to interconnect miniaturized human intestine, liver, brain, and kidney equivalents. All four organ models were pre-differentiated from iPSCs from the same healthy donor and integrated into the microphysiological system. This model platform will allow the development of autologous co-culture cross-talk assays, disease induction, and subsequent drug testing.
	Brull et al., 2020	Incorporation of stem cell-derived astrocytes into neuronal organoids to allow neuro-glial	In this publication, a fast and robust method to generate 3D cultured human dopaminergic neurons (LUHMES) for toxicity testing and long-term culture was presented. These cultures, which can be stably incorporated with human stem cell-derived

		interactions in toxicological studies	astrocytes or microglia, offer new approaches to quantify toxicant effects on organoids by standard technology and high throughput.
New test systems	Kobolak et al., 2020	Human induced pluripotent stem cell-derived 3D-neurospheres are suitable for neurotoxicity screening	Human iPSC-derived 3D neurospheres were exposed to different well-known toxicants with or without neurotoxic effect and examined at various stages of the differentiation with an ATP-based cell viability assay optimized for 3D-tissues. The acute exposure to different classes of toxicants revealed distinct susceptibility profiles in a differentiation stage-dependent manner. This indicates that hiPSC-based 3D *in vitro* neurosphere models could be used effectively to evaluate neurotoxicity and can be developed further to detect developmental neurotoxicity and thus replace or complement the use of animal models in various basic research and pharmaceutical applications.
	Ballester et al., 2019	Direct conversion of human fibroblast to hepatocytes using a single inducible polycistronic vector	A strategy was described to generate an unlimited source of homogeneously induced hepatocyte-like cells from different genetic background donors, capable of performing typical hepatic functions suitable for drug research and other *in vitro* applications. Human fibroblasts were reprogrammed into induced hepatocyte-like cells through the expression of a set of transcription factors via lentiviral vector.
	Coll et al., 2018	Generation of hepatic stellate cells from human pluripotent stem cells enables *in vitro* modelling of liver fibrosis.	The authors reported a novel differentiation protocol to obtain hepatic stellate cell (HSC)-like cells from induced pluripotent stem cells (iPSC). Such iPSC-HSCs closely resemble primary human HSCs at the transcriptional, cellular, and functional levels. This approach would provide a robust *in vitro* system for studying HSC development, modelling liver fibrosis, and drug toxicity screening.
High Throughput Testing	Copple et al., 2019	Characterisation of the NRF2 transcriptional network and its response to chemical insult in primary human hepatocytes: Implications for prediction of drug-induced liver injury	Microarray technology was applied to perform weighted gene co-expression network analysis and explore perturbations of the Nrf2 transcriptional network. A major understanding of the pathway will improve the prediction of clinical toxicities such as drug-induced liver injury.
	Delp et al., 2019	Development of a neurotoxicity assay that is tuned to detect mitochondrial toxicants	The authors described the development of a neurotoxicity assay, specifically tailored to detect mitochondrial toxicants. The combination of the high throughput neurotoxicity screening assay with the mechanistic follow up of target site identification allowed both, more sensitive detection of neurotoxicants and a sharper definition of the mode of action of mitochondrial toxicants.
	Limonciel et al., 2018b	Persistence of epigenomic effects after recovery from repeated treatment with two nephrocarcinogens	Organ toxicity in the kidney was addressed. In this publication, the relevance of epigenetic regulation has been investigated as a putative mechanism of long-lasting effects of chemicals. Nephrocarcinogens were tested on the human proximal tubule cell line

			RPTEC/TERT1 using high-content mRNA microarrays coupled with miRNA, histone acetylation and DNA methylation arrays and metabolomics. The integration of omics datasets suggested that the epigenetic mechanisms investigated were not the driving forces in the gene expression changes induced by the chemicals.
	Limonciel et al., 2018a	Comparison of base-line and chemical-induced transcriptomic responses in heparg and RPTEC/TERT1cells using TempO-Seq	The use of gene expression profiling for toxicity assessment was also described by, applied to kidney and liver toxicity models. Transcriptomic alterations were evaluated in differentiated kidney (RPTEC/TERT1) and liver (HepaRG) cells and compared to non-transcriptomic label-free sensitive endpoints of chemical-induced disturbances. The results showed that utilizing a gene panel of about 3000 probes, it is possible to discriminate basal tissue-specific signatures, generate dose-response relationships, and discriminate compound-specific and cell type-specific responses.
	Ramirez et al., 2018	Prediction of liver toxicity and mode of action using metabolomics *in vitro* in HepG2 cells	New omics technologies can provide comprehensive information on the toxicological mode of action of compounds. In this publication, the authors describe how a combination of mass-spectroscopy metabolomics with an *in vitro* liver toxicity model can be used for identifying organ toxicity in a robust, reliable, human-relevant manner.
	Campos et al., 2020	Inflammation-associated suppression of metabolic gene networks in acute and chronic liver disease	Inflammation has been recognized as essential for restorative regeneration. This publication analysed the sequential processes during onset of liver injury and subsequent regeneration based on time-resolved transcriptional regulatory networks (TRNs) to understand the relationship between inflammation, mature organ function, and regeneration. Spatiotemporal investigation differentiated lobular zones for signalling and transcription factor expression.
	Schimming et al., 2019	System microscopy of stress response pathways in cholestasis research	Application of quantitative image analysis for prediction of cholestasis-inducing toxicants. The authors described the use of high-throughput live-cell visualization of GFP-tagged key proteins of the oxidative stress response/Nrf2 pathway and inflammatory cytokine signalling to follow the temporal responses of individual cells.
iPSC reporters	Wink et al., 2018	Dynamic imaging of adaptive stress response pathway activation for prediction of drug induced liver injury	Systematic evaluation of the application of four key adaptive stress pathway reporters for the prediction of DILI liability: SRXN1-GFP (oxidative stress), CHOP-GFP (ER stress/UPR response), p21 (p53-mediated DNA damage-related response) and ICAM1 (NF-κB-mediated inflammatory signalling). 118 FDA-labelled drugs in five human exposure relevant concentrations were evaluated for reporter activation using live-cell confocal imaging.
	Yang et al., 2020	ATF 6 is a critical determinant of chop	In this study, microscopy-based quantification and dynamic modelling were used to detect the

		dynamics during the unfolded protein response.	molecular actors (ATF6) central in the activation of adaptive stress responses in HepG2 reporter cell lines.
iPSC reporters/ HTT	Bischoff et al., 2019	A systematic analysis of Nrf2 pathway activation dynamics during repeated xenobiotic exposure	Innovative technologies such as high content imaging and high-throughput transcriptome analysis have been applied to investigate pathways of toxicity. An example is represented by the Nrf2 pathway and its response to chemical insult. The authors made use of single-cell live imaging to quantitatively monitor the dynamics of the Nrf2 pathway during repeated toxic exposure, taking advantage of engineered fluorescent protein reporter cell lines.
Test strategy/AOP	Gu et al., 2018	Relevance of the incubation period in cytotoxicity testing with primary human hepatocytes	The publication addresses a central scientific topic of EU-ToxRisk: how long do *in vitro* experiments need to run to predict human-repeated-dose toxicity? The authors explored how the variation of the incubation period of a test compound can significantly influence the results of *in vitro* tests. Different treatment periods were compared to identify test conditions that would better correspond to human repeated-dose toxicity.
	Leist et al., 2017	Adverse Outcome Pathways: Opportunities, Limitations and Open Questions	The history of the AOP concept and its most prominent strengths are discussed, including the advantages of a formalized approach, the systematic collection of weight-of-evidence, the linkage of mechanisms to apical endpoints, the examination of the plausibility of epidemiological data, the identification of critical knowledge gaps and the design of mechanistic test methods. Potential weaknesses and shortcomings of the AOP rule set are addressed.
	Escher et al., 2019	Towards Grouping Concepts Based on New Approach Methodologies in Chemical Hazard Assessment: The Read-Across Approach of the EU-ToxRisk Project	Outlining a general RAx assessment concept using NAMs to support hazard characterization of grouped compounds by generating data on their dynamic and kinetic properties. Also outlining how mechanistic knowledge such as AOPs can be utilized. Describes the use of toxicokinetic models, in vitro to in vivo extrapolation, and anchoring to the existing in vivo data.
	Nyffeler et al., 2018	A structure-activity relationship linking non-planar PCBs to functional deficits of neural crest cells: new roles for connexins	Migration of neural crest cells (NCC) is a fundamental developmental process, and test methods to identify interfering toxicants have been developed. The authors explored whether the establishment of two overlapping structure-activity relationships (SAR)-linking chemical structure on the one hand to a phenotypic test outcome, and on the other hand to a mechanistic endpoint-was useful as a strategy to identify relevant toxicity mechanisms. For this purpose, polychlorinated biphenyls (PCB) were investigated as a case study.
	Krebs et al., 2020	The EU-ToxRisk Method Documentation, Data Processing and Chemical	Hazard assessment, based on new approach methods (NAM), requires the use of batteries of assays, where individual tests may be contributed by

		Testing Pipeline for the Regulatory Use of New Approach Methods	different laboratories. A unified strategy, as established by the EU-ToxRisk project, is presented. It details all procedures required to allow test information to be used for integrated hazard assessment, strategic project decisions and/or for regulatory purposes.
	Terron et al., 2018	An adverse outcome pathway for parkinsonian motor deficits associated with mitochondrial complex I inhibition	The authors describe a novel AOP demonstrating the mechanistic plausibility for epidemiological observations on a relationship between pesticide exposure and an elevated risk for Parkinson's disease development.
	Maertens et al., 2018	Weighted gene correlation network analysis (WGCNA) reveals novel transcription factors associated with bisphenol a dose-response	The authors explored the strategy to integrate additional tools, required for omics-based data analysis and visualization, into the AOP Knowledge Base, a main repository for AOPs, making use of WikiPathways.<br>The authors showed how this interoperability allows the needed integration of omics data linked to the molecular pathways with AOPs. Moreover, they demonstrate how this approach will improve risk assessment, because omics data will be linked directly to key events and therefore allow the comprehensive understanding and description of AOPs.
Test strategy	Albrecht et al., 2019	Prediction of human drug-induced liver injury (DILI) in relation to oral doses and blood concentrations	In the publication, a novel *in vitro/in silico* method to predicting the risk of human drug-induced liver injury in relation to oral doses and patient blood concentrations was described. The performance of the *in vitro* system was optimized by the application of two novel test performance metrics: the toxicity separation index, which quantifies how well a test differentiates between hepatotoxic and non-hepatotoxic compounds; and the toxicity estimation index, which measures how well hepatotoxic blood concentrations *in vivo* can be estimated.

Computational toxicology	Hemmerich et al., 2020	Using machine learning methods and structural alerts for prediction of mitochondrial toxicity	Despite well-established assays, such as the seahorse and glucose/galactose assay, an *in silico* approach to mitochondrial toxicity is still feasible, particularly when it comes to the assessment of large compound libraries. Therefore, *in silico* approaches could be very beneficial to indicate hazards early in the drug development pipeline. By combining multiple endpoints, we derived the largest so far published dataset on mitochondrial toxicity. A thorough data analysis shows that molecules causing mitochondrial toxicity can be distinguished by physicochemical properties. Finally, the combination of machine learning and structural alerts highlights the suitability for *in silico* risk assessment of mitochondrial toxicity.
	Troger et al., 2020	Identification of mitochondrial toxicants by combined *in silico* and *in vitro* studies – a	Drugs that modulate mitochondrial function can cause severe adverse effects. Unfortunately, mitochondrial toxicity is often not detected in animal models, which stresses the need for predictive *in*

		structure-based view on the adverse outcome pathway	*silico* approaches. In this study, we present a model for predicting mitochondrial toxicity focusing on human mitochondrial respiratory complex I inhibition by combining structure-based methods with machine learning. These results demonstrate that risk assessment and hazard analysis can benefit from combining structure-based methods and machine learning.
	Luechtefeld et al., 2018	Machine Learning of Toxicological Big Data Enables Read-Across Structure Activity Relationships (RASAR) Outperforming Animal Test Reproducibility	Describing a novel *in silico* approach called RASAR (read-across structure-activity relationship) which uses novel computational tools to define chemical similarity. Simple RASAR models tested in cross-validation achieve 70%-80% balanced accuracies. By combining RASAR models across toxicological domains (e.g. skin sensitization and skin irritation) balanced accuracies of 80%-95% were reached
	Gadaleta et al., 2018a	A new semi-automated workflow for chemical data retrieval and quality checking for modeling applications	An example of the application of a quality assurance workflow to *in silico* QSAR tools has been described by. The authors have designed a semi-automated workflow to integrate structural data retrieval, automated data comparison, chemical structure cleaning, and data selection and standardization. Application of such quality assurance procedure is critical to properly use QSAR tools and to use them to explore complex endpoints.
	Gadaleta et al., 2018b	QSAR modeling of ToxCast assays relevant to the molecular initiating events of AOPs leading to hepatic steatosis.	As described in another recent publication by the same group, a QSAR modeling approach has in fact been utilized to explore the adverse outcome pathways underlying induction of hepatic steatosis, by prediction of its molecular initiating events.
	Zgheib et al., 2019	Application of three approaches for quantitative AOP development to renal toxicity	Quantitative AOPs (qAOPs) providing dose-time-response predictions would be valuable for risk assessment. In their work, they compared three approaches for qAOP building: empirical dose-response modelling, Bayesian network calibration, and systems biology modelling, applying them to the quantification of a simplified oxidative stress-induced chronic kidney disease AOP.
ADME	Fisher et al., 2019 / Toma et al., 2018	Vivd: Virtual *in vitro* distribution model for the mechanistic prediction of intracellular concentrations of chemicals in *in vitro* toxicity assays/ QSAR development for plasma protein binding: Influence of the ionization state	Computational approaches for modelling the intracellular concentrations have been worked out. *In vitro* distribution models were developed to predict the freely dissolved concentrations, taking also into account differential ionization of test compounds between the media and cell cytoplasm. These models could improve *in vitro*-to-*in vivo* extrapolation of toxicity endpoints by determining intracellular concentrations for a more accurate translation to *in vivo*.
	Simeon et al., 2020	Development of a generic zebrafish embryo PBPK	In order to better explain, predict, or extrapolate to humans the developmental toxicity effects of

		model and application to the developmental toxicity assessment of valproic acid analogs	chemicals to zebrafish embryos, the authors developed a physiologically-based pharmacokinetic (PBPK) model designed to predict organ concentrations of neutral or ionizable chemicals, up to 120 h post-fertilization. *Ab initio* model predictions were established on data obtained on culture medium and embryo concentrations of valproic acid and nine analogues during continuous dosing under the OECD test guideline 236. The use of target organ concentrations substantially shifted the magnitude of dose-response parameters and the relative toxicity ranking of chemicals studied.
	Toropov and Toropova, 2017	The index of ideality of correlation: A criterion of predictive potential of QSPR/QSAR models?	The *in silico* side of the project is exemplified by a QSAR publication on blood-brain barrier transport of pesticides, and by prediction of LAT-1 transporter substrates, which is also important for drug and toxicant penetration into the brain.
	Toropova et al., 2018	The application of new hard-descriptor available from the coral software to building up NOAEL models.	Continuous QSAR models have been developed and validated for the prediction of no-observed-adverse-effect (NOAEL) in rats, using training and test sets from the Fraunhofer RepDose® database and EFSA's Chemical Hazards Database: OpenFoodTox. This paper demonstrates that the HARD index, as an integrated attribute of SMILES, improves the prediction power of NOAEL values using the continuous QSAR models and Monte Carlo simulations.
Risk assessment infrastructure	Rovida et al., 2020	Internationalization of read-across as a validated new approach method (NAM) for regulatory toxicology	The decision to prepare a review on the state of the art of RAx as a tool for risk assessment for regulatory purposes was taken during a workshop with international experts in Ranco, Italy in July 2018. Three major issues were identified that need optimization to allow a higher regulatory acceptance rate of the RAx procedure: (i) the definition of similarity of source and target, (ii) the translation of biological/toxicological activity of source to target, in the RAx procedure, and (iii) how to deal with issues of ADME that may differ between source and target. The use of new approach methodologies (NAM) was discussed as one of the most important innovations to improve the acceptability of RAx.
	Krebs et al., 2019	Template for the Description of Cell-Based Toxicological Test Methods to Allow Evaluation and Regulatory Use of the Data	This paper introduces the details and the practical application of an annotated toxicity test method template (ToxTemp) which was developed by the EU-ToxRisk project. The ToxTemp comprises all requirements of OECD Guidance Document 211 (GD211) on method documentation. It gives broad space to the inclusion of acceptance criteria for test elements, and a comprehensive and transparent definition of the test system. Such a template was endorsed by more than 30 experts from industry, regulatory bodies, and academia to improve the quality of the developed NAM.

	Graepel et al., 2019	Paradigm shift in safety assessment using new approach methods: The EU-ToxRisk strategy	The EU-ToxRisk research project is an interdisciplinary research project that aims to advance the paradigm shift in toxicology towards NAM-based approaches for risk assessment. In this European research project, experts in the fields of *in vitro* and *in silico* techniques and risk assessment from academia, industry, and regulatory agencies work together. Using a series of custom-designed case studies, the EU-ToxRisk battery of NAMs is being evaluated to learn how to carry out safety assessment using NAMs. This review article provides an overview of the project, its aims, its approach, and the methodologies that are being used.
	Busquet et al., 2020	Harnessing the power of novel animal-free test methods for the development of COVID-19 drugs and vaccines	The publication describes how past investments in NAMs for drug safety, efficacy, and quality evaluation can be leveraged for speedy drug discovery regarding COVID-19. Argues the importance of diversification in drug discovery strategies towards non-animal alternative approaches.
	Bal-Price et al., 2018	Recommendation on test readiness criteria for new approach methods in toxicology: Exemplified for developmental neurotoxicity	Multiple non-animal-based test methods have never been formally validated. In order to use such NAMs in a regulatory context, criteria to define their readiness are necessary. Readiness criteria, compiled during a stakeholder workshop, uniting scientists from academia, industry, and regulatory authorities are presented.

Supplementary Table 2: Top-10 EU-ToxRisk publications ranked by citation rate

Author (year)	Title	Description	Number of citations*
Leist et al. (2017)	Adverse outcome pathways: opportunities, limitations and open questions.	The review covers the history of the AOP concept and its most prominent strengths (formalized approach, the systematic collection of weight of evidence, the linkage of mechanisms to apical end points, etc.). Exemplary toxicological studies are also presented to discuss the linearity assumptions of AOP and its practical implementation.	105
Jansen et al. (2017)[#]	The ascending pathophysiology of cholestatic liver disease.	The review highlights the physiopathological mechanisms leading to the development of cholestatic liver disease. This review is meant to serve as a call to prioritize the development of biomarkers that help to obtain a better stratification of these diseases.	88
Ball et al. (2016)[#]	Toward Good Read-Across Practice (GRAP) Guidance.	This report presents the state of the art of read-across approach, summarizing learnings from reviewing ECHA published decisions regarding the relative successes/pitfalls surrounding read-across under REACH. The report comprehensively collects available existing tools and approaches, as the use of biological support data.	78
Toropov and Toropova (2017)	The index of ideality of correlation: A criterion of predictive potential of QSPR/QSAR models?	A QSAR publication on blood-brain barrier transport of pesticides, and prediction of LAT-1 transporter substrates, which is also important for drug and toxicant penetration into the brain.	55
Chandrasekaran et al. (2016)[#]	Astrocyte Differentiation of Human Pluripotent Stem Cells: New Tools for Neurological Disorder Research.	The review compares and summarizes the currently available protocols and strategies to generate human astrocytes from PSCs, focusing on the potential role of human-induced PSCs derived astrocytes in disease modelling.	54
Luechtefeld et al. (2018)	Machine Learning of Toxicological Big Data Enables Read-Across Structure Activity Relationships (RASAR) Outperforming Animal Test Reproducibility.	The publication describes a novel *in silico* approach called RASAR (read-across structure activity relationship) which uses novel computational tools to define chemical similarity. Combination of RASAR models across toxicological domains (e.g. skin sensitization and skin irritation) determined the achievements of 80%-95% balanced accuracies.	53
Bopp et al. (2018)[#]	Current EU research activities on combined exposure to multiple chemicals.	The publication collects contributions from the most relevant EU research projects (including EU-ToxRisk) on the experience and tools for the assessment of chemical mixtures toxicity.	45
Pamies et al. (2017)[#]	Good Cell Culture Practice for Stem Cells and Stem-Cell-Derived Models.	The workshop report addresses the need for an update of the first guidance on Good Cell Culture Practice (GCCP). Recommendations and novel principles were proposed to address the use of novel technologies as stem cells and stem-cell-derived models.	44
Toropova and Toropov (2017)[#]	The index of ideality of correlation: A criterion of predictability of QSAR	This original paper proposes the inclusion of new criterion of the predictive potential of quantitative structure–property/activity relationships (QSPRs/QSARs). The utilization of novel in silico	42

	models for skin permeability?	approach showed to improve the predictive potential of QSAR models.	
Schmidt et al. (2017)#	In Vitro acute and developmental Neurotoxicity Screening - an Overview of Cellular Platforms and High-throughput Technical.	This review aims to summarize the main cellular characteristics underlying neurotoxicity, and to offer an overview of cellular platforms and test methods to assess distinct parts of acute and developmental neurotoxicology.	41

*Citation rates were calculated using Plum Analytics (developed by Elsevier) as per July 16, 2020.

These publications have been co-funded by more than two other projects.

Supplementary Table 3: Top-10 EU-ToxRisk publications ranked by journal impact factor

Author (year)	Title	Description	Journal impact factor*
Coll et al. (2018)[#]	Generation of Hepatic Stellate Cells from Human Pluripotent Stem Cells Enables In Vitro Modeling of Liver Fibrosis.	The publication describes a novel differentiation protocol to obtain hepatic stellate cell (HSC)-like cells from induced pluripotent stem cells (iPSC). Such iPSC-HSCs, closely resembling primary human HSCs at the transcriptional, cellular, and functional levels, would provide a robust *in vitro* system for modelling liver fibrosis, and performing drug toxicity screening.	23.3 (Cell Stem Cell)
Jansen et al. (2017)[#]	The ascending pathophysiology of cholestatic liver disease	See above (Supp. table 2)	14.1 (Hepatology)
Goldring et al. (2017)[#]	Stem cell–derived models to improve mechanistic understanding and prediction of human drug-induced liver injury.	This review collects the expertise of a number of researchers (including EU-ToxRisk project's coordinator) to address the application of stem cells in hepatotoxicity safety assessment, and to make recommendations for the way forward. The publication strongly highlights the importance of benchmarking stem cell-derived hepatocyte-like cells to their human counterparts for chemical safety assessment purposes.	14.1 (Hepatology)
Ghallab et al. (2019)[#]	Bile Microinfarcts in Cholestasis Are Initiated by Rupture of the Apical Hepatocyte Membrane and Cause Shunting of Bile to Sinusoidal Blood.	Taking into consideration an AOP concept developed by the EU-ToxRisk project, this publication elucidates novel pathophysiological mechanisms leading to Charcot–Gombault necrosis (biliary infarcts due to late complication of extrahepatic cholestasis).	14.1 (Hepatology)
Sarkans et al. (2018)[#]	The BioStudies database—one stop shop for all data supporting a life sciences study.	The publication focuses on the description of the public database BioStudies (used by the EU-ToxRisk project) for long-term storage of multi-omics data.	11.6 (Nucleic Acids Research)
Sachinidis et al. (2019)	Road map for development of stem cell-based alternative test methods	The publication identifies limiting factors and recommendations for further refinement of differentiation protocols for hiPSCs to hepatocyte-like cells. The authors present a road map to facilitate the development and assess the performance of test systems.	11.0 (Trends in Molecular Medicine)
Gutbier et al. (2018)	Prevention of neuronal apoptosis by astrocytes through thiol-mediated stress response modulation and accelerated recovery from proteotoxic stress.	An advanced organ-specific assay was established and described. A well-established model of postmitotic human dopaminergic neurons (LUHMES cells) was used in the absence or in co-culture with astrocytes to investigate the mechanisms involved in the endogenous neuro-defensive activity of astrocytes.	8.0 (Cell death and Differentiatio n)
Bopp et al. (2018) [#]	Current EU research activities on combined exposure to multiple chemicals.	See above (Supp. table 2)	7.9 (Environment International)
Benfenati et al. (2019)	Integrating in silico models and read-across methods for predicting toxicity of	The authors discuss a framework on weight of evidence (as published by EFSA) to assess	7.9 (Environment International)

	chemicals: A step-wise strategy.	systematic integration of results or values obtained from *in silico* models and read-across.	
Drakvik et al. (2020)#	Statement on advancing the assessment of chemical mixtures and their risks for human health and the environment.	The workshop report highlights the main conclusion from the workshop on “Advancing the Assessment of Chemical Mixtures and their Risks for Human Health and the Environment” co-organized by the Joint Research Center in Ispra. Gaps, key messages and future research needs are presented.	7.9 (Environment International)

* The indicated impact factors are the scores assigned to the journals at the publication year.

# These publications have been co-funded by more than two other projects.

Supplementary references

- Albrecht, W., Kappenberg, F., Brecklinghaus, T. et al. (2019). Prediction of human drug-induced liver injury (DILI) in relation to oral doses and blood concentrations. *Arch Toxicol 93*, 1609-1637. doi:10.1007/s00204-019-02492-9
- Ball, N., Cronin, M. T., Shen, J. et al. (2016). Toward good read-across practice (grap) guidance. ALTEX 33, 149-166. doi:10.14573/altex.1601251
- Ballester, M., Bolonio, M., Santamaria, R. et al. (2019). Direct conversion of human fibroblast to hepatocytes using a single inducible polycistronic vector. *Stem Cell Res Ther 10*, 317. doi:10.1186/s13287-019-1416-5
- Bal-Price, A., Hogberg, H. T., Crofton, K. M. et al. (2018). Recommendation on test readiness criteria for new approach methods in toxicology: Exemplified for developmental neurotoxicity. *ALTEX 35*, 306-352. doi:10.14573/altex.1712081
- Benfenati, E., Chaudhry, Q., Gini, G. et al. (2019). Integrating in silico models and read-across methods for predicting toxicity of chemicals: A step-wise strategy. Environ Int 131, 105060. doi:10.1016/j.envint.2019.105060
- Birnbaum, L. S., Burke, T. A. and Jones, J. J. (2016). Informing 21st-century risk assessments with 21st-century science. *Environ Health Perspect 124*, A60-63. doi:10.1289/ehp.1511135
- Bischoff, L. J. M., Kuijper, I. A., Schimming, J. P. et al. (2019). A systematic analysis of nrf2 pathway activation dynamics during repeated xenobiotic exposure. *Arch Toxicol 93*, 435-451. doi:10.1007/s00204-018-2353-2
- Bopp, S. K., Barouki, R., Brack, W. et al. (2018). Current eu research activities on combined exposure to multiple chemicals. Environ Int 120, 544-562. doi:10.1016/j.envint.2018.07.037
- Brull, M., Spreng, A. S., Gutbier, S. et al. (2020). Incorporation of stem cell-derived astrocytes into neuronal organoids to allow neuro-glial interactions in toxicological studies. *ALTEX* doi:10.14573/altex.1911111
- Busquet, F., Hartung, T., Pallocca, G. et al. (2020). Harnessing the power of novel animal-free test methods for the development of COVID-19 drugs and vaccines. *Arch Toxicol* doi:10.1007/s00204-020-02787-2
- Campos, G., Schmidt-Heck, W., De Smedt, J. et al. (2020). Inflammation-associated suppression of metabolic gene networks in acute and chronic liver disease. *Arch Toxicol 94*, 205-217. doi:10.1007/s00204-019-02630-3
- Chandrasekaran, A., Avci, H. X., Leist, M. et al. (2016). Astrocyte differentiation of human pluripotent stem cells: New tools for neurological disorder research. Front Cell Neurosci 10, 215. doi:10.3389/fncel.2016.00215
- Coll, M., Perea, L., Boon, R. et al. (2018). Generation of hepatic stellate cells from human pluripotent stem cells enables in vitro modeling of liver fibrosis. *Cell Stem Cell 23*, 101-113 e107. doi:10.1016/j.stem.2018.05.027
- Collins, F. S., Gray, G. M. and Bucher, J. R. (2008). Toxicology. Transforming environmental health protection. *Science 319*, 906-907. doi:10.1126/science.1154619
- Copple, I. M., den Hollander, W., Callegaro, G. et al. (2019). Characterisation of the nrf2 transcriptional network and its response to chemical insult in primary human hepatocytes: Implications for prediction of drug-induced liver injury. *Arch Toxicol 93*, 385-399. doi:10.1007/s00204-018-2354-1
- Cote, I., Andersen, M. E., Ankley, G. T. et al. (2016). The next generation of risk assessment multi-year study-highlights of findings, applications to risk assessment, and future directions. *Environ Health Perspect 124*, 1671-1682. doi:10.1289/EHP233
- Daneshian, M., Kamp, H., Hengstler, J. et al. (2016). Highlight report: Launch of a large integrated european in vitro toxicology project: Eu-toxrisk. *Arch Toxicol 90*, 1021-1024. doi:10.1007/s00204-016-1698-7
- Delp, J., Funke, M., Rudolf, F. et al. (2019). Development of a neurotoxicity assay that is tuned to detect mitochondrial toxicants. *Arch Toxicol 93*, 1585-1608. doi:10.1007/s00204-019-02473-y
- Drakvik, E., Altenburger, R., Aoki, Y. et al. (2020). Statement on advancing the assessment of chemical mixtures and their risks for human health and the environment. Environ Int 134, 105267. doi:10.1016/j.envint.2019.105267
- Dreser, N., Madjar, K., Holzer, A. K. et al. (2020). Development of a neural rosette formation assay (RoFa) to identify neurodevelopmental toxicants and to characterize their transcriptome disturbances. *Arch Toxicol 94*, 151-171. doi:10.1007/s00204-019-02612-5
- Embry, M. R., Bachman, A. N., Bell, D. R. et al. (2014). Risk assessment in the 21st century: Roadmap and matrix. *Crit Rev Toxicol 44 Suppl 3*, 6-16. doi:10.3109/10408444.2014.931924
- Escher, S. E., Kamp, H., Bennekou, S. H. et al. (2019). Towards grouping concepts based on new approach methodologies in chemical hazard assessment: The read-across approach of the EU-ToxRisk project. *Arch Toxicol 93*, 3643-3667. doi:10.1007/s00204-019-02591-7
- Fisher, C., Simeon, S., Jamei, M. et al. (2019). Vivd: Virtual in vitro distribution model for the mechanistic prediction of intracellular concentrations of chemicals in in vitro toxicity assays. *Toxicol In Vitro 58*, 42-50. doi:10.1016/j.tiv.2018.12.017

- Gadaleta, D., Lombardo, A., Toma, C. et al. (2018a). A new semi-automated workflow for chemical data retrieval and quality checking for modeling applications. *J Cheminform 10*, 60. doi:10.1186/s13321-018-0315-6
- Gadaleta, D., Manganelli, S., Roncaglioni, A. et al. (2018b). Qsar modeling of Toxcast assays relevant to the molecular initiating events of AOPs leading to hepatic steatosis. *J Chem Inf Model 58*, 1501-1517. doi:10.1021/acs.jcim.8b00297
- Ghallab, A., Hofmann, U., Sezgin, S. et al. (2019). Bile microinfarcts in cholestasis are initiated by rupture of the apical hepatocyte membrane and cause shunting of bile to sinusoidal blood. Hepatology 69, 666-683. doi:10.1002/hep.30213
- Gocht, T., Berggren, E., Ahr, H. J. et al. (2015). The seurat-1 approach towards animal free human safety assessment. *ALTEX 32*, 9-24. doi:10.14573/altex.1408041
- Goldring, C., Antoine, D. J., Bonner, F. et al. (2017). Stem cell-derived models to improve mechanistic understanding and prediction of human drug-induced liver injury. Hepatology 65, 710-721. doi:10.1002/hep.28886
- Graepel, R., ter Braak, B., Escher, S. E. et al. (2019). Paradigm shift in safety assessment using new approach methods: The EU-ToxRisk strategy. *Current Opinion in Toxicology 15*, 33-39. doi:10.1016/j.cotox.2019.03.005
- Gu, X., Albrecht, W., Edlund, K. et al. (2018). Relevance of the incubation period in cytotoxicity testing with primary human hepatocytes. *Arch Toxicol 92*, 3505-3515. doi:10.1007/s00204-018-2302-0
- Gutbier, S., Spreng, A. S., Delp, J. et al. (2018). Prevention of neuronal apoptosis by astrocytes through thiol-mediated stress response modulation and accelerated recovery from proteotoxic stress. Cell Death Differ 25, 2101-2117. doi:10.1038/s41418-018-0229-x
- Hemmerich, J., Troger, F., Fuzi, B. et al. (2020). Using machine learning methods and structural alerts for prediction of mitochondrial toxicity. *Mol Inform 39*, e2000005. doi:10.1002/minf.202000005
- Hiemstra, S., Ramaiahgari, S. C., Wink, S. et al. (2019). High-throughput confocal imaging of differentiated 3D liver-like spheroid cellular stress response reporters for identification of drug-induced liver injury liability. *Arch Toxicol 93*, 2895-2911. doi:10.1007/s00204-019-02552-0
- Jansen, P. L., Ghallab, A., Vartak, N. et al. (2017). The ascending pathophysiology of cholestatic liver disease. Hepatology 65, 722-738. doi:10.1002/hep.28965
- Juberg, D. R., Knudsen, T. B., Sander, M. et al. (2017). Futuretox iii: Bridges for translation. *Toxicol Sci 155*, 22-31. doi:10.1093/toxsci/kfw194
- Kobolak, J., Teglasi, A., Bellak, T. et al. (2020). Human-induced pluripotent stem cell-derived 3d-neurospheres are suitable for neurotoxicity screening. *Cells 9*, doi:10.3390/cells9051122
- Krebs, A., Waldmann, T., Wilks, M. F. et al. (2019). Template for the description of cell-based toxicological test methods to allow evaluation and regulatory use of the data. *ALTEX 36*, 682-699. doi:10.14573/altex.1909271
- Krebs, A., van Vugt-Lussenburg, B. M. A., Waldmann, T. et al. (2020). The EU-ToxRisk method documentation, data processing and chemical testing pipeline for the regulatory use of new approach methods. *Arch Toxicol* doi:10.1007/s00204-020-02802-6
- Leist, M., Hartung, T. and Nicotera, P. (2008). The dawning of a new age of toxicology. *ALTEX 25*, 103-114.
- Leist, M., Lidbury, B. A., Yang, C. et al. (2012). Novel technologies and an overall strategy to allow hazard assessment and risk prediction of chemicals, cosmetics, and drugs with animal-free methods. *ALTEX 29*, 373-388. doi:10.14573/altex.2012.4.373
- Leist, M., Hasiwa, N., Rovida, C. et al. (2014). Consensus report on the future of animal-free systemic toxicity testing. *ALTEX 31*, 341-356. doi:10.14573/altex.1406091
- Leist, M., Ghallab, A., Graepel, R. et al. (2017). Adverse outcome pathways: Opportunities, limitations and open questions. *Arch Toxicol 91*, 3477-3505. doi:10.1007/s00204-017-2045-3
- Limonciel, A., Ates, G., Carta, G. et al. (2018a). Comparison of base-line and chemical-induced transcriptomic responses in HepaRG and RPTEC/tert1 cells using tempo-seq. *Arch Toxicol 92*, 2517-2531. doi:10.1007/s00204-018-2256-2
- Limonciel, A., van Breda, S. G., Jiang, X. et al. (2018b). Persistence of epigenomic effects after recovery from repeated treatment with two nephrocarcinogens. *Front Genet 9*, 558. doi:10.3389/fgene.2018.00558
- Luechtefeld, T., Marsh, D., Rowlands, C. et al. (2018). Machine learning of toxicological big data enables read-across structure activity relationships (RASAR) outperforming animal test reproducibility. *Toxicol Sci 165*, 198-212. doi:10.1093/toxsci/kfy152

- Maertens, A., Tran, V., Kleensang, A. et al. (2018). Weighted gene correlation network analysis (WGNA) reveals novel transcription factors associated with bisphenol-a dose-response. *Front Genet 9*, 508. doi:10.3389/fgene.2018.00508
- Nyffeler, J., Chovancova, P., Dolde, X. et al. (2018). A structure-activity relationship linking non-planar PCBs to functional deficits of neural crest cells: New roles for connexins. *Arch Toxicol 92*, 1225-1247. doi:10.1007/s00204-017-2125-4
- Paini, A., Leonard, J. A., Joossens, E. et al. (2019). Next generation physiologically based kinetic (ng-pbk) models in support of regulatory decision making. *Comput Toxicol 9*, 61-72. doi:10.1016/j.comtox.2018.11.002
- Pamies, D., Bal-Price, A., Simeonov, A. et al. (2017). Good cell culture practice for stem cells and stem-cell-derived models. ALTEX 34, 95-132. doi:10.14573/altex.1607121
- Pastoor, T. P., Bachman, A. N., Bell, D. R. et al. (2014). A 21st century roadmap for human health risk assessment. *Crit Rev Toxicol 44 Suppl 3*, 1-5. doi:10.3109/10408444.2014.931923
- Ramirez, T., Strigun, A., Verlohner, A. et al. (2018). Prediction of liver toxicity and mode of action using metabolomics in vitro in hepg2 cells. *Arch Toxicol 92*, 893-906. doi:10.1007/s00204-017-2079-6
- Ramme, A. P., Koenig, L., Hasenberg, T. et al. (2019). Autologous induced pluripotent stem cell-derived four-organ-chip. *Future Sci OA 5*, FSO413. doi:10.2144/fsoa-2019-0065
- Rovida, C., Barton-Maclaren, T., Benfenati, E. et al. (2020). Internationalization of read-across as a validated new approach method (NAM) for regulatory toxicology. *ALTEX* doi:10.14573/altex.1912181
- Sachinidis, A., Albrecht, W., Nell, P. et al. (2019). Road map for development of stem cell-based alternative test methods. Trends Mol Med 25, 470-481. doi:10.1016/j.molmed.2019.04.003
- Sarkans, U., Gostev, M., Athar, A. et al. (2018). The biostudies database-one stop shop for all data supporting a life sciences study. Nucleic Acids Res 46, D1266-D1270. doi:10.1093/nar/gkx965
- Schimming, J. P., Ter Braak, B., Niemeijer, M. et al. (2019). System microscopy of stress response pathways in cholestasis research. *Methods Mol Biol 1981*, 187-202. doi:10.1007/978-1-4939-9420-5_13
- Schmidt, B. Z., Lehmann, M., Gutbier, S. et al. (2017). In vitro acute and developmental neurotoxicity screening: An overview of cellular platforms and high-throughput technical possibilities. Arch Toxicol 91, 1-33. doi:10.1007/s00204-016-1805-9
- Simeon, S., Brotzmann, K., Fisher, C. et al. (2020). Development of a generic zebrafish embryo PBPK model and application to the developmental toxicity assessment of valproic acid analogs. *Reprod Toxicol 93*, 219-229. doi:10.1016/j.reprotox.2020.02.010
- Terron, A., Bal-Price, A., Paini, A. et al. (2018). An adverse outcome pathway for parkinsonian motor deficits associated with mitochondrial complex I inhibition. *Arch Toxicol 92*, 41-82. doi:10.1007/s00204-017-2133-4
- Tice, R. R., Austin, C. P., Kavlock, R. J. et al. (2013). Improving the human hazard characterization of chemicals: A tox21 update. *Environ Health Perspect 121*, 756-765. doi:10.1289/ehp.1205784
- Toma, C., Gadaleta, D., Roncaglioni, A. et al. (2018). QSAR development for plasma protein binding: Influence of the ionization state. *Pharm Res 36*, 28. doi:10.1007/s11095-018-2561-8
- Toropov, A. A. and Toropova, A. P. (2017). The index of ideality of correlation: A criterion of predictive potential of QSPR/QSAR models? *Mutat Res 819*, 31-37. doi:10.1016/j.mrgentox.2017.05.008
- Toropova, A. P. and Toropov, A. A. (2017). The index of ideality of correlation: A criterion of predictability of QSAR models for skin permeability? Sci Total Environ 586, 466-472. doi:10.1016/j.scitotenv.2017.01.198
- Toropova, A. P., Toropov, A. A., Marzo, M. et al. (2018). The application of new hard-descriptor available from the coral software to building up NOAEL models. *Food Chem Toxicol 112*, 544-550. doi:10.1016/j.fct.2017.03.060
- Troger, F., Delp, J., Funke, M. et al. (2020). Identification of mitochondrial toxicants by combined in silico and in vitro studies – a structure-based view on the adverse outcome pathway. *Computational Toxicology 14*, 100-123. doi:10.1016/j.comtox.2020.100123
- Wink, S., Hiemstra, S. W., Huppelschoten, S. et al. (2018). Dynamic imaging of adaptive stress response pathway activation for prediction of drug induced liver injury. *Arch Toxicol 92*, 1797-1814. doi:10.1007/s00204-018-2178-z
- Wolf, D. C., Bachman, A., Barrett, G. et al. (2016). Illustrative case using the risk21 roadmap and matrix: Prioritization for evaluation of chemicals found in drinking water. *Crit Rev Toxicol 46*, 43-53. doi:10.3109/10408444.2015.1082973
- Yang, H., Niemeijer, M., van de Water, B. et al. (2020). Atf6 is a critical determinant of chop dynamics during the unfolded protein response. *iScience 23*, 100860. doi:10.1016/j.isci.2020.100860
- Zgheib, E., Gao, W., Limonciel, A. et al. (2019). Application of three approaches for quantitative AOP development to renal toxicity. *Computational Toxicology 11*, 1-13. doi:10.1016/j.comtox.2019.02.001
